# A Rare Case of Pericardial Involvement in Pseudomyxoma Peritonei

**DOI:** 10.7759/cureus.97196

**Published:** 2025-11-18

**Authors:** Edoardo Brogiolo, Lilia Lagha

**Affiliations:** 1 Department of Medicine, Wexham Park Hospital, Frimley Health NHS Foundation Trust, Slough, GBR

**Keywords:** case report, cytoreductive surgery (crs), hyperthermic perioperative chemotherapy (hipec), pericardial effusion, pericarditis, pmp, pseudomyxoma peritonei

## Abstract

Pseudomyxoma peritonei (PMP) is an uncommon clinical entity characterised by the progressive accumulation of mucinous ascites within the peritoneal cavity. We describe the case of a 59-year-old man with recurrent PMP secondary to an appendiceal mucinous neoplasm, who presented with acute chest pain and dyspnoea. Investigations revealed transient constrictive pericarditis with mild pericardial effusion and associated precardiac lymphadenopathy. Management with anti-inflammatory therapy led to clinical and echocardiographic resolution of pericardial inflammation. This case highlights an association between PMP and pericardial involvement, emphasising the importance of early echocardiographic evaluation in PMP patients presenting with chest pain. Recognising this rare association may guide timely and appropriate management, preventing the development of potentially life-threatening cardiac tamponade.

## Introduction

Pseudomyxoma peritonei (PMP) is a disease characterised by the intraperitoneal accumulation of mucinous ascites. This mucinous invasion with ascites and peritoneal implants originates from a perforated mucinous neoplasm, most commonly of appendiceal origin, albeit the ovaries and colorectal region ​[1]​ have also been implicated. It disseminates via the so-called *redistribution phenomenon*​ [2]​ along the normal flow of peritoneal fluid​, reaching predictable anatomical sites including the greater omentum, left abdominal gutter, right under-hemidiaphragm, and the pelvis​ [1]. It is a rare condition, with as few as 2-3 cases per million inhabitants estimated to occur every year in the United Kingdom​ [3]​. PMP is a radiological diagnosis, with contrast CT showing loculated mucinous collections coating peritoneal surfaces, with scalloping of visceral organs and omental caking​ [4]. The current standard of care​ involves a combination of cytoreductive surgery (CRS) combined with intra-abdominal irrigation with heated cytotoxic chemotherapy, also known as hyperthermic intraperitoneal chemotherapy (HIPEC) [5]​. While not known for yielding distant metastases, untreated PMP remains a locally aggressive disease process​ [1]​, with a slow yet relentless course ultimately leading to the patient’s demise from cachexia, bowel obstruction, and other complications of intra-abdominal organ compression [6]. Metastatic spread to extra-abdominal sites, including the pericardium, is exceedingly uncommon [7]. However, at least one case of metastatic spread of adenocarcinomatous cells from a PMP to the pericardium, with consequent tamponade, has been portrayed in the literature​ [8]​. In this case report, we will describe an association between PMP and the development of pericardial inflammation and effusion, in the absence of any other potential aetiological contributor. We will also be highlighting the importance of considering acute echocardiography in the management of this type of patient presenting with chest pain. 

## Case presentation

A 59-year-old man presented to the hospital complaining of central chest pain radiating to the back, associated with dyspnoea. His past medical history included hypertension, type 2 diabetes mellitus, chronic kidney disease stage 3, and ischaemic heart disease with a previous myocardial infarction. He also had a background of PMP, originating from a primary appendiceal mucinous neoplasm. This had been initially treated two years earlier in accordance with the NICE UK guidelines​ [5]​ using the Sugarbaker technique, comprising intra-abdominal CRS (total colectomy, omentectomy, and ileostomy formation) and HIPEC. The procedure was successful, but surveillance imaging a few weeks before this presentation demonstrated radiological evidence of disease recurrence. 

**Figure 1:**
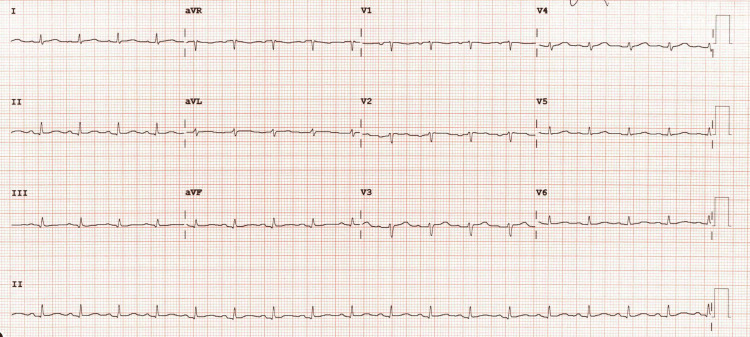
Admission ECG showing sinus tachycardia

At the time of the current admission, his chest pain had developed insidiously over the course of approximately 12 hours. The pain was described as a tight band across his chest radiating posteriorly, exacerbated by lying supine or on the left side. On physical examination, tachycardia and bilateral basal inspiratory crackles were noted. Electrocardiography (Figure 1, Video 1) revealed low-voltage sinus tachycardia, and daily serial troponin measurements remained within normal limits (9 ng/L, 4 ng/L, <4 ng/L). Rapid diagnostic testing for respiratory viral pathogens (influenza, SARS-CoV-2, RSV) was negative.

**Video 1:** Echocardiogram showing pericardial effusion next to the right ventricular outflow tract

**Figure 2:**
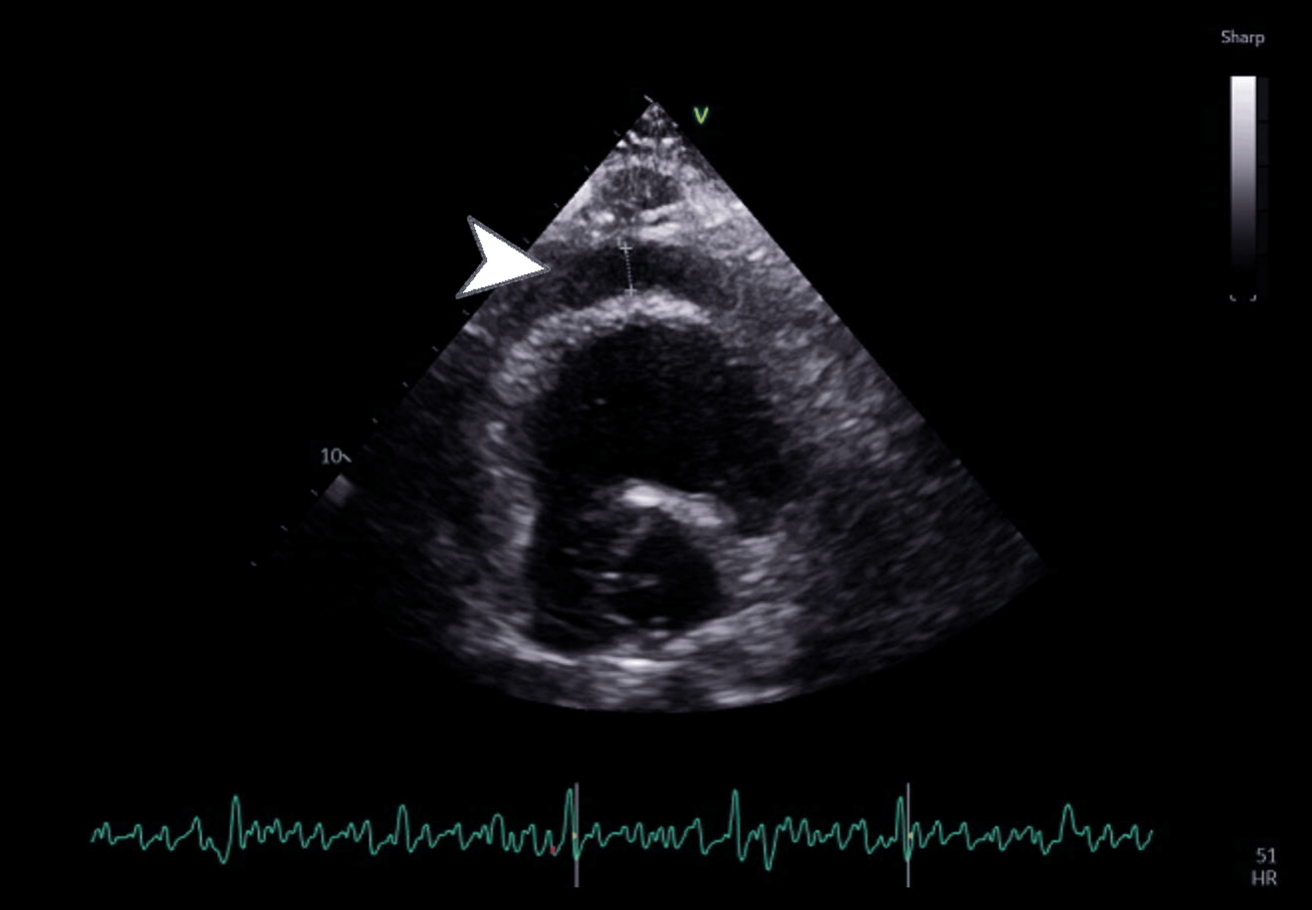
Echocardiogram showing pericardial effusion next to the right ventricular outflow tract (still frame)

**Figure 3:**
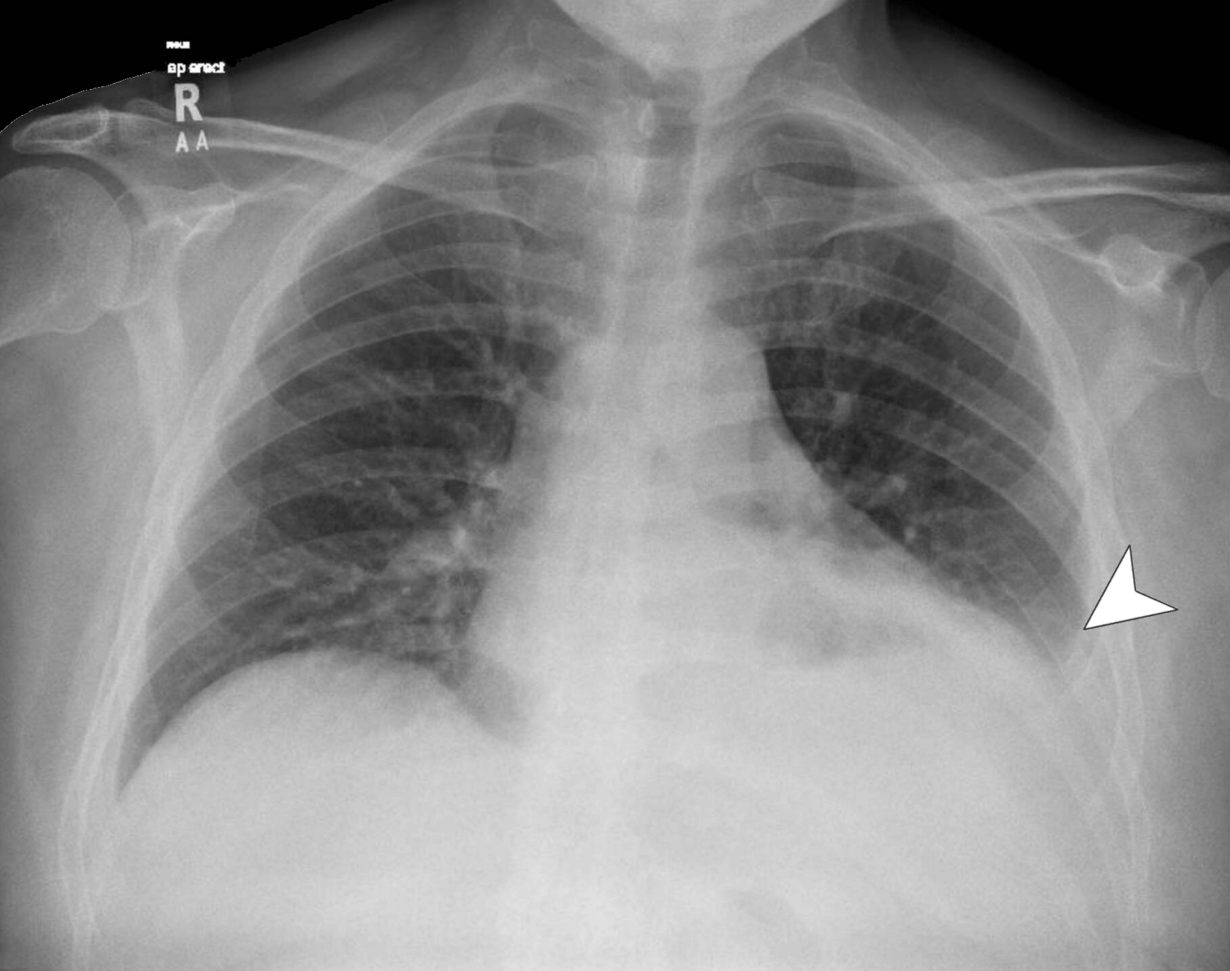
Chest X-ray showing left-sided pleural effusion

Point-of-care ultrasound assessment in the emergency department, and subsequent formal transthoracic echocardiography, highlighted a mild pericardial effusion near the anterior right ventricular outflow tract (Figure 2), measuring between 0.7 and 1.0 cm in depth, and therefore not amenable to pericardiocentesis. Left ventricular ejection fraction was visually estimated at 55-60%, with normal wall thickness and impaired diastolic function for age. No regional wall motion abnormalities were observed. Mitral E/A of >0.8 (1.62), a dilated inferior vena cava at 3.5 cm with poor respiratory collapse, septal shift on inspiration, and mitral medial e' velocity of >8 cm/s (11 cm/s) completed a picture suggestive of constrictive pericarditis. A large left-sided pleural effusion, corresponding to chest radiography (Figure 3) findings, was also observed. Conservative treatment was started with intravenous fluids, colchicine, and analgesia. 

**Figure 4:**
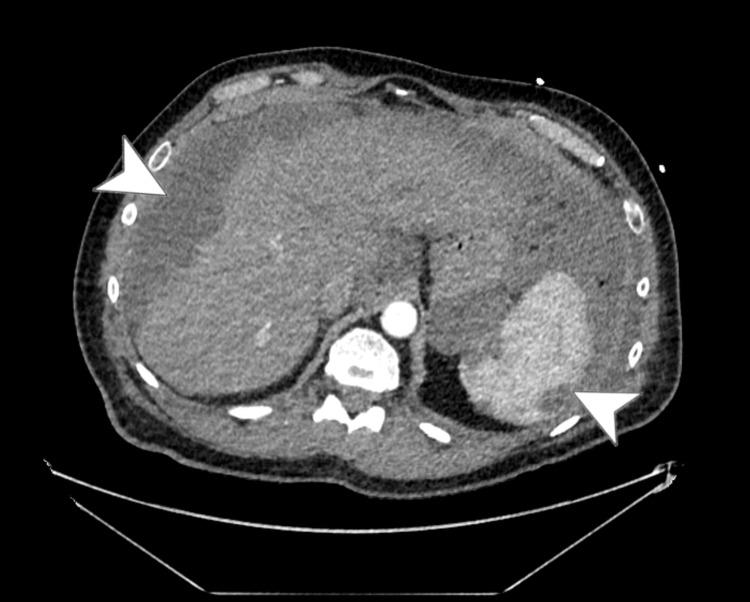
Axial CT view showing scalloping of the liver (left arrowhead) and spleen (right arrowhead)

**Figure 5:**
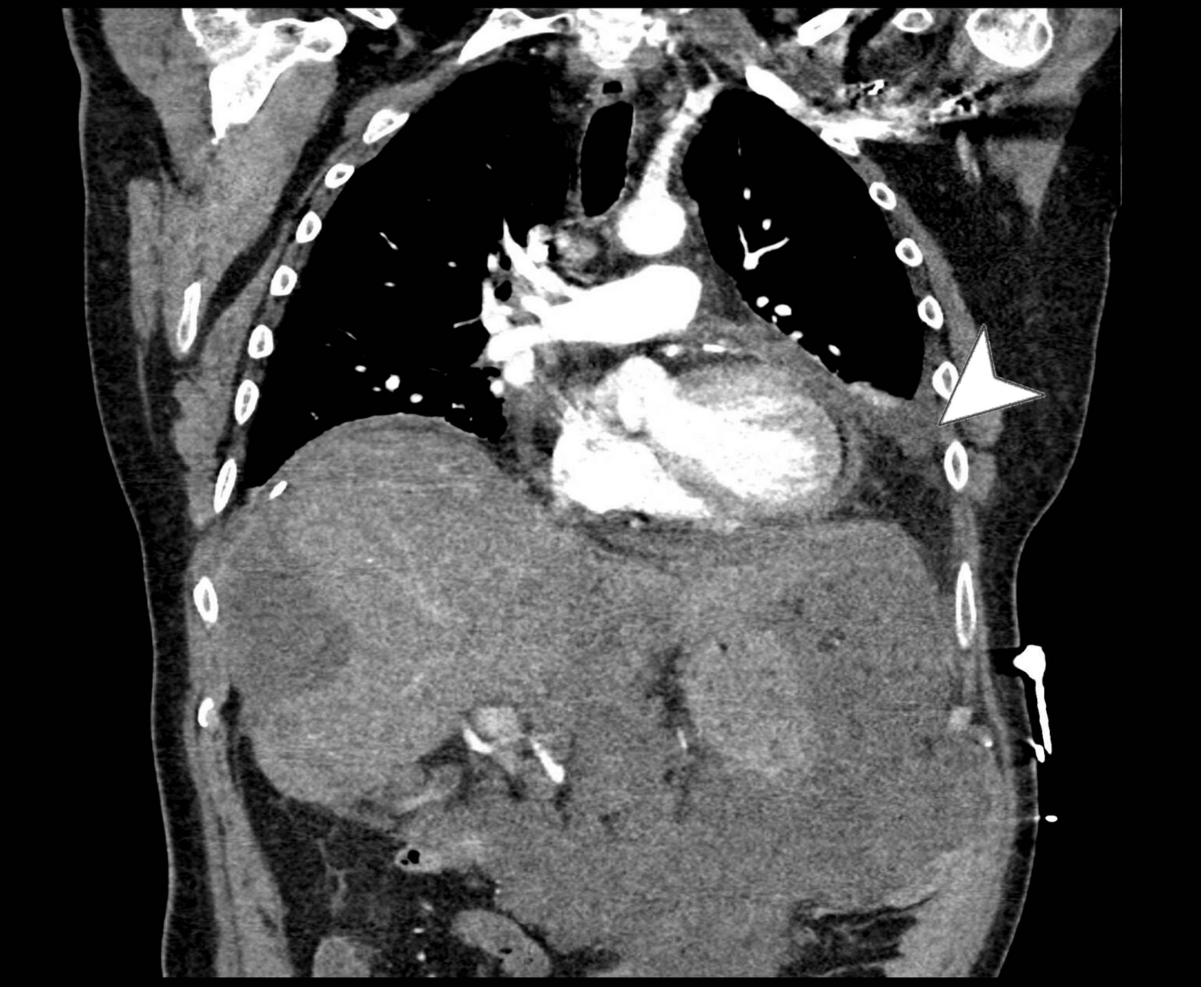
Coronal CT view showing left-sided organised pleural effusion

On day 2, further evaluation with CT imaging of the chest, abdomen, and pelvis revealed characteristic scalloping of the hepatic and splenic surfaces (Figure 4), consistent with PMP, and confirmed a mild pericardial effusion, 1.6 cm at its maximum depth. Prominent precardiac lymph nodes were detected, measuring up to 12 mm in their short axis. Bilateral pleural effusions, worse and loculated on the left, were confirmed on CT (Figure 5). 

Urgent left-sided thoracocentesis was performed on day 4, which drained 1500 mL of pleural fluid, relieving his respiratory symptoms. The pleural effusion appeared yellow, non-bloody, and rich in white blood cells under the microscope. A cytological study confirmed the presence of mixed inflammatory cells, prompting treatment with empirical antibiotic therapy; no malignant cells were found on either cytological or immunohistochemical analysis. The patient was subsequently reviewed by the Acute Oncology Team, and ongoing follow-up with specialist cancer services was arranged. Medical management of the pericarditis led to resolution of chest pain. Repeat focused echocardiography on day 6 (Video 2, Video 3) demonstrated improvement of the pericardial effusion (down to 0.23-0.30 cm), with resolution of the features of constrictive pericarditis, no observable septal shift, and normalisation of the inferior vena cava diameter (≤21 mm). The patient was discharged with planned outpatient follow-up in cardiology, respiratory, and oncology clinics. 

**Video 2:** Echocardiogram showing improvement of the pericardial effusion and no observable septal shift

**Video 3:** Echocardiogram showing inferior vena cava diameter < 21 mm

## Discussion

Pericardial involvement in PMP represents an exceptionally rare manifestation of an already uncommon pathological entity, which typically remains confined to the peritoneal cavity but may, in rare instances, extend beyond its expected anatomical boundaries​ [2-7]​. This case contributes to the limited body of literature documenting cardiac complications associated with PMP [8]. Although definitive diagnostic confirmation through pericardiocentesis and cytological analysis of pericardial fluid was not possible, the concurrent presence of pericardial effusion, pericardial inflammation, and precardiac lymphadenopathy raises the possibility that either inflammatory extension via transdiaphragmatic lymphatic pathways or direct metastatic dissemination may have contributed to the observed pathology​ [9]​. This is further supported by the temporal relationship between the recurrence of PMP and pericardial inflammation and by the concurrent development of asymmetrical pleural effusions. 

Clinically, pericardial effusion and inflammation in such patients can easily be misattributed to cardiac or infective causes, particularly given the non-specific nature of chest pain and dyspnoea in individuals with complex multimorbidity [10]. The use of point-of-care and formal echocardiography was crucial in identifying pericardial effusion and constrictive physiology, allowing for the timely initiation of conservative therapy and avoidance of unnecessary invasive procedures; echocardiography is widely recognised as the first-line imaging modality for pericardial effusion and hemodynamic assessment in suspected tamponade or constriction​ [9-11]​. The coexistence of pleural and pericardial effusions, recurrent intra-abdominal disease, and regional lymphadenopathy highlight the potential for PMP to produce systemic or transcompartmental effects not traditionally recognised as part of its disease spectrum. 

Recognition of such extra-abdominal presentations is clinically significant for two main reasons. First, pericardial inflammation and effusion can present with non-specific symptoms [12] that mimic other cardiovascular diseases such as acute coronary syndromes or pulmonary embolism. Without timely recognition, progression to cardiac tamponade is a possible life-threatening outcome: malignant pleural effusions are a known aetiology of tamponade ​[10]​. Second, this case reinforces the value of early echocardiographic assessment as a diagnostic adjunct in PMP patients presenting with atypical chest pain or dyspnoea, enabling quick, appropriate intervention.

## Conclusions

Pericardial involvement in PMP represents an exceedingly rare clinical entity. In this case, we witnessed that pericardial inflammation and effusion may arise in association with PMP. Early recognition through echocardiographic assessment is vital, as pericardial effusion can mimic other acute cardiovascular presentations and may progress to life-threatening tamponade if unrecognised. Prompt echocardiographic evaluation and early institution of anti-inflammatory therapy can prevent progression to haemodynamic compromise. As this phenomenon is exceedingly rare, further reports and long-term follow-up studies are warranted to better delineate the underlying mechanisms and prognostic implications.
